# Left ventricular contractile reserve by stress echocardiography as a predictor of response to cardiac resynchronization therapy in heart failure: a systematic review and meta-analysis

**DOI:** 10.1186/s12872-017-0657-4

**Published:** 2017-08-16

**Authors:** Quirino Ciampi, Clara Carpeggiani, Claudio Michelassi, Bruno Villari, Eugenio Picano

**Affiliations:** 1 Division of Cardiology, Fatebenefratelli Hospital, Viale Principe di Napoli, 12, I-82100 Benevento, Italy; 2 CNR, Institute of Clinical Physiology, Pisa, Italy

**Keywords:** Cardiac resynchronization therapy, Contractile reserve, Stress echo, Heart failure

## Abstract

**Background:**

The presence of left ventricular contractile reserve (LVCR) during stress echo (SE) may provide favorable response to cardiac resynchronization therapy (CRT) in heart failure patients. The aim of the study was to perform a meta-analysis of available SE data in this set of patients.

**Methods:**

From a Pubmed and Advance Google Scholar database web based search scan up to December 2016, we initially identified 5906 records. From this initial set, we removed that did not include SE and duplicate studies. We assessed for eligibility 71 full-text articles assessed for eligibility, and 60 of them did not meet the inclusion criteria as follow: 1) heart failure patients with NYHA class III and IV, depressed ejection fraction (EF <35%) and QRS duration ≥120 ms at study entry; 2) SE with assessment of LVCR; 3) Follow-up data. LVCR during SE was identified as reduction in wall motion score index and/or an increase in EF.

**Results:**

Eleven studies with 861 patients (mean age 67 ± 9 years, ejection fraction 25 ± 6%) were included in the meta-analysis. The type of stress was either exercise (*n* = 2) or dobutamine (*n* = 9), the latter with low-dose (10 mcg) in two, intermediate-dose (20 mcg) in five, and high-dose (40 mcg) protocol in two studies. LVCR was detected in 555 patients (63%) and CRT-response was present in 584 (66%). The overall odds ratio for LVCR to predict a favorable CRT response was 2.06 (95%, CI 1.70–2-43), Z score: 11.055, *p* < 0.001.

**Conclusion:**

The presence of LVCR during SE with either dobutamine or exercise is associated with a greater chance of response to CRT. This parameter is now ready to be tested in a prospective multicenter trial to select patients more likely to benefit from CRT.

## Background

Cardiac resynchronization therapy (CRT) is a frequently used effective therapy in patients with heart failure, characterized by high cost, significant risks and relatively high rate of non-response [1–3]. Therefore, the identification of responders using noninvasive tools prior to implantation is an important, yet challenging, task [4]. QRS width remains the only cornerstone for CRT indication in severely symptomatic heart failure patients with reduced ejection fraction. [4].

The 2016 ESC guidelines do not mention the role of left ventricle myocardial contractile reserve (LVCR) during stress echocardiography (SE) [4], which can be useful for predicting outcomes of several medical and surgical interventions in patients with dilated cardiomyopathy of different etiology [5]. The 2016 joint recommendations of the American Society of Echocardiography (ASE) and European Association of Cardiovascular Imaging (EACVI) suggest that the absence of LVCR is a strong determinant of outcome and a potential marker of response to CRT [6].

The aim of the study was to assess the evidence base underlying the possible usefulness of LVCR in CRT patients by performing a meta-analysis of available SE data.

## Methods

### Search approach and appraisal of studies

Studies were found using Pubmed and Advance Google Scholar database web based search. The key terms used were: CRT, SE with dobutamine or exercise. When more than one study came from the same group, only the largest and most recent study was considered for inclusion.

### Inclusion criteria

We considered for inclusion if they evaluated the use of LVCR and viability evaluated using pharmacological or exercise stress echocardiography in regard to response after CRT.

The inclusion criteria were:

1) human studies with participants of any age requiring CRT for any indication, according to guidelines criteria [4]: heart failure patients with NYHA class III and IV, depressed ejection fraction (EF < 35%) and QRS duration ≥120 ms;2) CRT and SE were performed on the same population of patients; 3) parameters of LVCR before and after CRT implantation were reported; 4) presence of at least 6 months of follow-up.

### Exclusion criteria

Two blinded reviewers evaluated the records. Studies with only either LVCR or CRT data, case reports, case series, letters and editorials were excluded but relevant reviews were retrieved to identify additional studies. Full-text articles that did not meet inclusion criteria were excluded from final analysis.

### Data abstraction

**Fig. 1:**
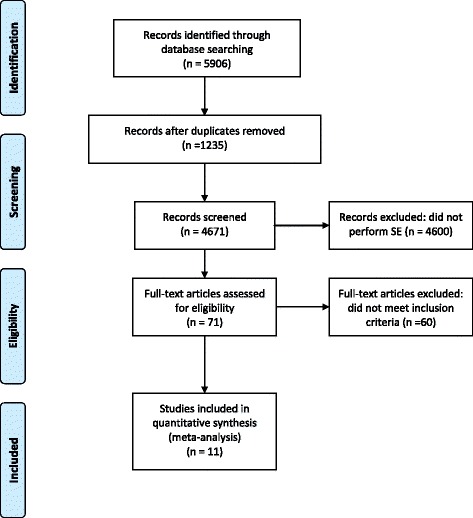
Study selection process

According to the Preferred Reporting Items for Systematic reviews and Meta-Analyses statement [7], from a Pubmed and Advance Google Scholar database web based search scan up to December 2016, we initially identified 5906 records, using the key terms CRT (cardiac resynchronization therapy) (5906 citations) AND SE (71 citations) AND dobutamine (19 citations) OR exercise echocardiography (7 citations). Of these records we removed the duplicate (1235 record) and the studies that did not perform SE (4600 records). Full-text articles assessed for eligibility were 71, screened to control whether the publications met the inclusion criteria. These inclusion criteria we identified 11 studies for analysis [8–18] (Fig. 1).

The key data elements that were extracted from each study were categorized as study characteristics, patient characteristics, treatment characteristics, and clinical outcome. Specifically, the main characteristics considered for each study were: first author’s name, journal and year of publication, duration of follow-up, number of patients, type of intervention, type of stress and dosage of stressor (Tables 1 and 2).

**Table 1:** Clinical characteristics of the studies included in the analysis

	Patients (n.)	Age (years)	Sex (male, %)	Ischemic etiology (%)	QRS duration (ms)	Follow-Up (months)	Responders CRT Criteria
Da Costa A [8]	67	70 ± 10	84%	34%	190 ± 28	12	clinical
Rocchi G [9]	64	64 ± 11	75%	31%	154 ± 25	6	echo/clinical
Ciampi Q [10]	59	70 ± 8	71%	55%	150 ± 27	11	echo/clinical
Lancellotti P [11]	51	70 ± 9	63%	67%	161 ± 25	6	echo/clinical
Senechal M [12]	49	66 ± 12	69%	69%	164 ± 30	6	echo/clinical
Chaundry FA [13]	54	69 ± 11	63%	54%	147 ± 20	7	echo/clinical
Altman RK [14]	31	68 ± 12	74%	65%	158 ± 22	24	echo/clinical
Gasparini M [15]	204	67 ± 10	70%	43%	150 ± 25	15	echo/clinical
Pugliese M [16]	102	71 ± 5	64%	87%	143 ± 11	6	echo/clinical
Mizia-Stec K [17]	129	62 ± 9	76%	48%	164 ± 24	12	echo/clinical
Murin P [18]	51	62 ± 8	75%	48%	150 ± 24	6	echo/clinical
Mean Value	78	67 ± 9	71%	55%	157 ± 24	10

**Table 2:** Rest and stress echocardiographic characteristics of the studies included in the analysis

	LVEDV at rest (ml)	LVESV at rest (ml)	LVEF at rest (%)	Type stressors	Dose (mcg)	LV Contractile reserve criteria
Da Costa A [8]	249 ± 92	187 ± 75	26 ± 5	dobutamine	10	EF increase >1.25-fold
Rocchi G [9]	183 ± 54	139 ± 45	24 ± 5	exercise	-	WMSI decrease >0.25
Ciampi Q [10]	176 ± 64	129 ± 52	27 ± 6	dobutamine	40	WMSI decrease >0.20
Lancellotti P [11]	184 ± 39	134 ± 32	27 ± 5	exercise	-	EF increase >6.5%
Senechal M [12]	216 ± 65	180 ± 62	19 ± 7	dobutamine	20	WMSI decrease >0.25
Chaundry FA [13]	108 ± 32	87 ± 23	18 ± 7	dobutamine	20	WMSI decrease >0.31
Altman RK [14]	-	116 ± 40	28 ± 6	dobutamine	10	EF increase >20%
Gasparini M [15]	215 ± 79	159 ± 65	27 ± 6	dobutamine	20	EF increase >5 points
Pugliese M [16]	-	-	27 ± 4	dobutamine	20	WMSI decrease >0.20
Mizia-Stec K [17]	-	-	24 ± 6	dobutamine	20	WMSI decrease >0.20
Murin P [18]	227 ± 66	168 ± 55	26 ± 7	dobutamine	40	EF increase >7%
Mean Value	195 ± 61	144 ± 49	25 ± 6		22	

For patients, the following variables were collected whenever available: sex; mean age; definition of significant disease; follow-up event; summary. In addition to these variables, estimates of sensitivity and specificity, and the absolute number of true-positive, false-negative, false-positive, and true-negative results were extracted per source study.

We did not contact authors to request additional information.

Various echocardiographic parameters were used to assess the LVCR identified eitheras a reduction in wall motion score index (WMSI, six studies) or an increase in ejection fraction (EF, five studies); in particular: decrease in WMSI >0.20 (three studies), >0.25 two studies) and >0.31 in one study; about EF: 1.5-fold increase (one study), >5 points (one study), >6.5% (one study), >7% (one study) and >20% (one study) (Table 2). Responders to CRT were identified on the basis of clinical (one study) or echocardiographic criteria (10 studies): clinical responders were defined as survivors who had a ≥ 1 grade improvement in NYHA class, and no new hospital admission for acute heart failure. Echocardiographic responders were defined as patients who showed decrease of LV end-systolic volume of at least 15% in 8 studies [9–12, 14, 16–18], decrease of LV end-systolic volume of at least 10% in 1 study [15] and improvement in LVEF of 5 points in 1 study [13].

### Data analysis

The pooled weighted estimation of sensitivity, specificity and accuracy are reported in Table 3. Calculations of sensitivity, specificity and accuracy were performed according to standard definitions.

**Table 3:** Contractile reserve to SE (CR+) and responders to CRT (CRT+) of the studies included in the analysis

	CR+ CRT+	CR+ CRT-	CR- CRT+	CR- CRT-	CR+	CRT+
Da Costa A [8]	28 (42%)	6 (9%)	19 (28%)	14 (21%)	34 (51%)	47 (70%)
Rocchi G [9]	41 (64%)	5 (8%)	2 (3%)	16 (25%)	46 (72%)	43 (67%)
Ciampi Q [10]	29 (49%)	13 (22%)	5 (8%)	12 (20%)	42 (71%)	34 (59%)
Lancellotti P [11]	27 (53%)	4 (8%)	3 (6%)	17 (33%)	31 (61%)	30 (59%)
Senechal M [12]	30 (61%)	1 (2%)	1 (2%)	17 (35%)	31 (63%)	31 (63%)
Chaundry FA [13]	29 (54%)	13 (24%)	2 (4%)	10 (19%)	42 (78%)	31 (58%)
Altman RK [14]	13 (42%)	7 (23%)	3 (10%)	8 (26%)	20 (65%)	16 (52%)
Gasparini M [15]	144 (71%)	22(11%)	16 (8%)	22 (11%)	166 (81%)	160 (79%)
Pugliese M [16]	37 (36%)	14 (14%)	23 (23%)	28 (27%)	51 (50%)	60 (59%)
Mizia-Stec K [17]	62 (48%)	5 (4%)	42 (33%)	20 (16%)	67 (52%)	104 (81%)
Murin P [18]	22 (43%)	3 (6%)	6 (12%)	20 (39%)	25 (49%)	28 (55%)
Total value (%)	462 (52%)	93 (11%)	122 (14%)	184 (21%)	555 (63%)	584 (66%)

Meta-regression analysis with continuous and categorical parameters was conducted using the metafor package for R [19]. Estimates of the average effect and 95% confidence intervals (CIs) of different parameters on CRT response were calculated with a fixed [20] and random-effect model [21]. The heterogeneity was tested using the Cochran Q statistic (following the chi-squared distribution) and I-squared (I^2^) statistics Higgins [20], which describes the proportion of total variation explained by between-study variation instead of chance. Higher I^2^statistic values imply more heterogeneity between studies than would be expected by chance alone. When the test for heterogeneity was statistically significant the random-effect model was applied directly. The odds ratio was chosen as outcome measure. Individual odds ratio (ORs) were estimated as the cross-product of cell counts in the corresponding 2 × 2 table, with variance of natural logarithm (ln) of OR equal to the sum of the reciprocal cell counts.

We expressed continuous data as mean ± SD, and dichotomous variables as percentages. We considered statistically significant a *p*-value <0.05.

**Fig. 2:**
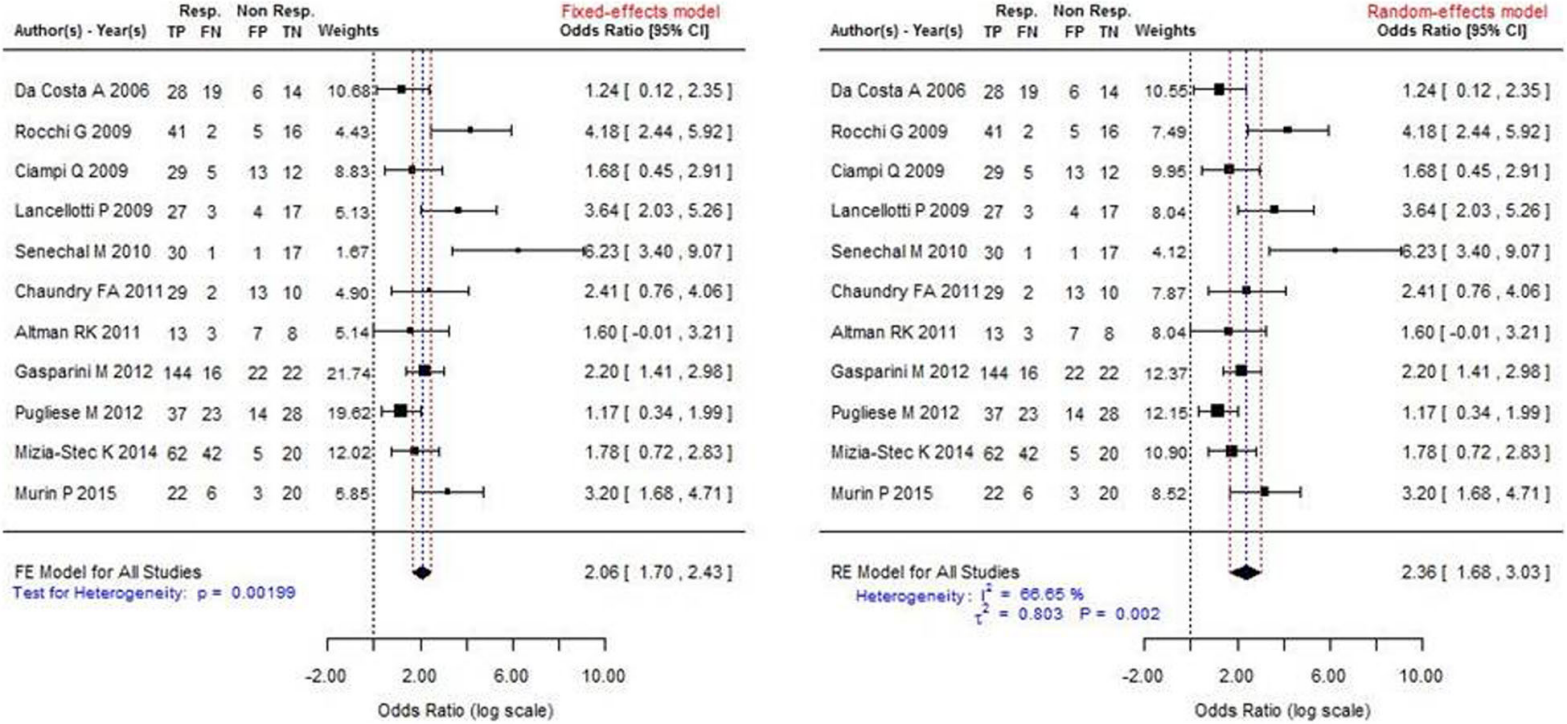
Forest plots showing the benefits associated with presence of LVCR. They include the same eleven studies, but in the left panel a fixed-effect analysis was used and in the right panel a random-effect analysis. Individual studies (identified by first author and reference number) are shown on the left, and their corresponding odds ratios (and confidence intervals) on the right. The area of each square is proportional to the weight in the final result. The measure effect is plotted as a diamond, and its lateral margins indicate confidence intervals for this estimate. Rocchi G and Lancellotti P studies used exercise, all the others dobutamine as stressor

**Fig. 3:**
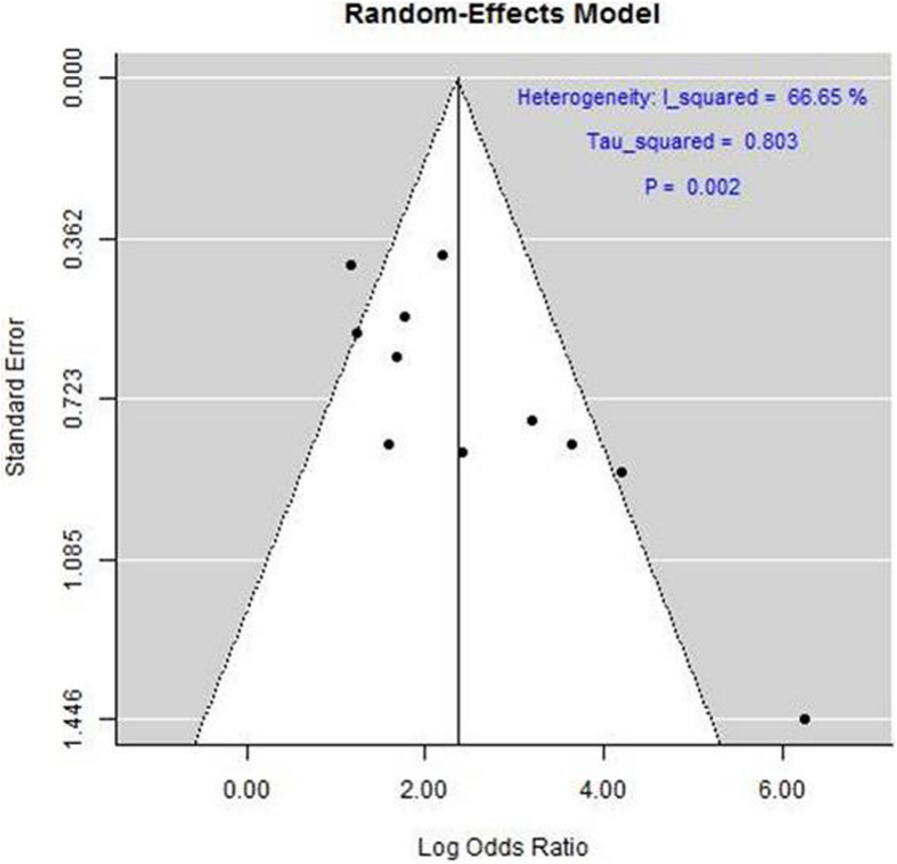
The funnel plot displaying log odds ratio against its standard error for each individual study

A graphical overview of the results was obtained by creating a forest plot [22] in which the observed effects were drawn proportional to the precision of the estimates (Fig. 2). Funnel plot was used for detecting heterogeneity (Fig. 3). The results are shown using a log scale for easier interpretation.

## Results

Eleven studies with 861 patients (mean age 67 ± 9 years, 71% male, 55% with ischemic cardiomyopathy) were included in the meta-analysis. Details of the studies and baseline clinical and echocardiographic characteristics are summarized in Tables 1 and 2. Resting ejection fraction was 25 ± 6%, QRS duration 157 ± 24 ms, end-diastolic and end-systolic volumes were 194 ± 61 ml and 144 ± 49 ml, respectively.

The type of stress was either exercise (*n* = 2) or dobutamine (*n* = 9), the latter with low-dose (up to 10 mcg) in two, intermediate-dose (up to 20 mcg) in five, and high-dose (40 mcg) protocol in two studies (Table 2).

In the analysis of 11studies, 555 patients (63%) showed LVCR (Table 3).

During mean follow-up of 10 months (range 6–24), 584 patients (66%) were responders to CRT, according to clinical and echocardiographic criteria (10 studies) or clinical criteria alone (one study).

In particular, we found the higher prevalence of patient responders to CRT in presence of LVCR (462/584:79%). The number of patients studied true positive with LVCR+ and responders to CRT was between a minimum of 36% and a maximum of 71%, with a mean value of 52%.The percentage of LVCR was between 49% and 81% with a mean value of 63%. The positive predictive value was 83% and negative predictive value was 60%.

The overall odds ratio for LVCR to predict a favorable CRT response was 2.06 (95%, CI 1.70–2-43), Z score: 11.055, *p* < 0.001. There was significant heterogeneity across the studies with a test of heterogeneity Q: 27.733, *p* = 0.00199 using fixed effect. Using random effect model we found the same results: the overall odds ratio for LVCR to predict a favorable CRT response was 2.36 (95%, CI 1.68–3.04), Z score: 6,84, *p* < 0.001 (Fig. 2). A significant heterogeneity across the studies was confirmed with a test of heterogeneity Q: 27.73, *p* = 0.002 (Figs. 2 and 3).

We also analyzed only the paper including only dobutamine test studies (*n* = 9). The overall odds ratio for LVCR to predict a favorable CRT response was 1.87 (95%, CI 1.48–2.25), test of heterogeneity Q: 17.37, *p* = 0.02.

No detectable difference across different LVCR criteria was found; in fact the overall odds ratio with WMSI was 2.58 (95%, CI 1.32–3.48), with a test of heterogeneity Q of 80.61%, *p* = 0.0018 and with EF as criteria of LVCR the overall odds ratio was 2.28 (95%, CI 1.46–3.10), with a test of heterogeneity Q of 52.45%, *p* = 0.087.

## Discussion

The presence of CR greatly increases the likelihood of response to CRT, and should probably be considered in the selection of suitable candidates. LVCR during SE identifies a group with a higher chance of functional recovery and clinical improvement after CRT. This might help the clinician in the still-elusive task of refining the criteria of identifying non-responders, which total about one-third of all patients with CRT, avoiding the attendant discomfort, risks and costs of implantation.

### Clinical implications

In the recent ESC guidelines [4] for the diagnosis and treatment of heart failure, ejection fraction, symptoms and QRS width remain the only criteria for identifying heart failure patients suitable for CRT, missed by imaging tests for dyssynchrony, shown to be of limited value for this challenging task. In addition, the ESC guidelines suggest that exercise or pharmacological stress echocardiography may be used for the assessment of myocardial ischemia and/or myocardial viability, but do not mention SE for identifying patients who will respond favorably to CRT [4].

Our results are consistent with previous paper published [23] and they corroborate the recommendations of EACVI and ASE on the clinical use of SE beyond coronary artery disease, which on the basis of a narrative review of the available literature concluded that “the absence of contractile reserve is a strong determinant of outcome and a potential marker of response to CRT” [6]. The present meta-analysis lends a more quantitative support to this recommendation mostly based on expert opinion. This finding shifts the focus from electrical (dyssynchrony) to the myocardial substrate of functional response. Similar information can be likely obtained with other techniques assessing myocardial viability with metabolic or structural or functional markers, such as nuclear imaging, or delayed enhancement or low-dose dobutamine cardiac magnetic resonance [24, 25]. In all these conditions, imaging of the metabolic, functional or structural integrity of the myocardium is a predictor of excellent response to medical interventions, such as medical therapy with beta-blockers [26], or interventions such as CRT [27] or coronary revascularization [28].

Prospective and extensive studies will serve to add CR as one of the criteria for CRT implantation in HF patients [29].

### Study limitations

The studies included in the meta-analysis showed some substantial heterogeneity. The studies included in the meta-analysis showed some substantial heterogeneity. The stresses employed were different (exercise and dobutamine); for the same stress, doses were different (for dobutamine, ranging from 10 to 40 mcg);even for the same stress at the same dose, the index parameters were different across the studies (ranging from WMSI to EF) and when the parameter was the same, different cut-offs were used in the different studies (Table 2). The major source of heterogeneity was the use of different stresses, exercise and dobutamine. In fact, the heterogeneity (measured by I2) was substantially decreased when only dobutamine studies were considered (I2 = 37.6%) compared to the overall analysis including the 2 exercise studies (I2 = 66.6%). When only dobutamine stress was considered, the data remained highly significant in spite of the reduced sample size, and with substantially reduced variability. The other source of heterogeneity was the echocardiographic parameter used to assess LVCR, since the heterogeneity was considerably higher considering studies based on WMSI (I2 = 80.6%) compared to studies with EF (I2 = 52.4%). We focused on very simple parameters based on WMSI or EF. These indices although appealingly simple suffer from some limitations, such as the qualitative and subjective nature of regional wall motion assessment and the pre-load and afterload- dependence of EF. Lastly, most studies did not warrant blinded assessment of CR and follow-up echocardiography.

We did not include more sophisticated or technologically advanced indices of left ventricular function such as left ventricular noninvasive pressure-volume relationship [30] or global longitudinal strain [31]: in particular global longitudinal strain might be more robust echo parameters in which to look for CR however, the inter-vendor variability and the high percentage of segments that can not be analyzed at rest and peak stress make this parameter still not fully usable for large-scale studies [32].

We also could not evaluate other, newer, promising measures of intra-ventricular dyssynchrony such as septal flash [33] or apical rocking [34] which may have incremental value over LVCR.

Recent studies have highlighted the importance of a multiparametric approach rather than an approach focusing on a single parameter in predicting CRT response. This applies to integrating echo parameters with non-echocardiographic parameters (such as scar on MRI imaging) and also combining different echocardiographic parameters (such as left ventricular contractile reserve with septal flash). To overcome some of these limitations, in the ongoing Stress echo 2020 study endorsed by the Italian Society of Echocardiography [29], a specific subproject will also address the subset with heart failure and reduced ejection fraction with CRT, prospectively evaluated with a standard stress echo approach (same test, same dose, same parameters) prior to intervention.

## Conclusion

The presence of LVCR during SE with either dobutamine or exercise is associated with greater chance of functional recovery after CRT. This parameter is now ready to be tested in a prospective multicenter trial to select patients more likely to benefit from CRT.

## Abbreviations

ASE: American Society of Echocardiography
CRT: Cardiac resynchronization therapy
EACVI: European Association of Cardiovascular Imaging
EF: Ejection fraction
ESC: European Society of Cardiology
LVCR: Left ventricular contractile reserve
LVEDV: Left ventricular end-diastolic volume
LVESV: Left ventricular end-systolic volume
NYHA functional class: New York Heart Association functional class
SE: Stress echocardiography
WMSI: Wall motion score index
